# Cytotoxic effects of solvent-extracted active components of *Salvia miltiorrhiza* Bunge on human cancer cell lines

**DOI:** 10.3892/etm.2015.2252

**Published:** 2015-02-03

**Authors:** BOKYUNG SUNG, HYE SUN CHUNG, MINJUNG KIM, YONG JUNG KANG, DONG HWAN KIM, SEONG YEON HWANG, MIN JO KIM, CHEOL MIN KIM, HAE YOUNG CHUNG, NAM DEUK KIM

**Affiliations:** 1 Department of Pharmacy, College of Pharmacy, Pusan National University, Busan 609-735, Republic of Korea; 2 Department of Biochemistry, School of Medicine, Pusan National University, Yangsan 626-770, Republic of Korea

**Keywords:** *Salvia miltiorrhiza* Bunge, extraction, tanshinones, cancer

## Abstract

Herbal extracts and dietary supplements may be extracted from the medicinal plants used in traditional Chinese medicine, and are used increasingly commonly worldwide for their benefits to health and quality of life. Thus, ensuring that they are safe for human consumption is a critical issue for the preparation of plant extracts as dietary supplements. The present study investigated extracts of *Salvia miltiorrhiza* Bunge (*S. miltiorrhiza*), traditionally used in Asian countries to treat a variety of conditions, as a dietary supplement or as an ingredient in functional foods. Dried *S. miltiorrhiza* root was extracted with various solvents and under varying extraction conditions, and the effects of the extracts on the viability of five human cancer cell lines were compared. Extracts obtained using 100% ethanol and 100% acetone as solvents exhibited more potent effects compared with extracts obtained using 70 and 30% aqueous ethanol. Furthermore, the active components of *S. miltiorrhiza* ethanol extracts*,* known as tanshinones, were investigated. Dihydrotanshinone I was observed to exhibit a higher cytotoxic potential compared with the other tanshinones in the majority of the examined cell lines. Conversely, cryptotanshinone exhibited weak anti-cancer activity. In summary, the results of the present study suggest that the active components obtained from an ethanol extract of *S. miltiorrhiza* possess the potential to be used as ingredients in functional and health care foods that may be used to improve the effectiveness of chemotherapeutics in the prevention and/or treatment of cancer.

## Introduction

The dried root of *Salvia miltiorrhiza* Bunge (*S. miltiorrhiza*; Radix Salvia Miltiorrhizae), known as Danshen in Chinese, Dansam in Korean and Tansen in Japanese, is one of the most commonly used substances in traditional Chinese medicine (TCM), and is also used in other Asian countries, including Korea and Japan (1). *S. miltiorrhiza* has been used in the treatment of a variety of conditions, including cardiovascular disease, cerebrovascular disease, diabetic vascular complications (1), liver dysfunction (2) and renal disease (3). Furthermore, Wen *et al* previously demonstrated that *S. miltiorrhiza* is effective in the treatment of gastrointestinal inflammatory disease (4). The active chemical constituents of *S. miltiorrhiza* root have been studied extensively. These active components, the majority of which have been identified and purified, are generally divided into two major groups; water-soluble phenolic compounds and lipophilic diterpene quinones (5). The lipid-soluble compounds, usually extracted using alcohol solvents, are rich in abietanoids and diterpene quinones (tanshinones). Numerous diterpenoid tanshinones have been extracted from *S. miltiorrhiza*, including dihydrotanshinone I, cryptotanshinone and tanshinones I and IIA, which are the three most commonly studied. These tanshinones have been reported to exhibit antioxidative (6–9), anti-inflammatory (6,10,11), anti-allergic (12) and anti-cancer effects (13,14).

In China, *S. miltiorrhiza* has been used as a medicine or dietary supplement for improving health. The oral administration of this medicinal herb appears to exhibit negligible effects on the pharmacokinetics of therapeutic agents, such as docetaxel or clopidogrel, indicating the potential benefits of combining *S. miltiorrhiza* with standard therapeutics (15). Furthermore, the combination of *S. miltiorrhiza* and its active constituents with other TCM or chemotherapeutic substances has been observed to result in more notable anti-cancer effects compared with either agent alone (16,17). However, the safety of orally administered *S. miltiorrhiza* as a nutritional or dietary supplement is not known and may be a potential disadvantage to its use as a therapeutic.

The first step in the utilization of medicinal plants as dietary supplements, health foods/nutritional supplements or pharmaceuticals is the extraction of the bioactive constituents from the plant materials. The active components of plant materials are commonly extracted using a solvent. Numerous solvents, including methanol, ethanol, acetone and ethyl acetate, have previously been used to prepare extracts from plant materials, either alone or in combination and at varying aqueous dilutions. Ethanol is considered to be a particularly effective solvent, and its use is permitted by the food industry for the preparation of dietary supplements or functional foods, as it is safe for human consumption (18). Numerous *S. miltiorrhiza* preparations are currently available, including lipophilic and hydrophilic extracts. The ethanol extract of *S. miltiorrhiza*, which is rich in lipophilic constituents, is the most commonly used in Chinese clinics (19).

The present study aimed to compare the effects of a number of ethanol extracts of *S. miltiorrhiza*, obtained using ethanol at various aqueous dilutions as a solvent, with an extract obtained using acetone, one of the most common solvents for hydrophobic compounds, on the viability of cancer cells. In addition, the cytotoxic effects of the bioactive constituents indicated to be present in the most active fraction of *S. miltiorrhiza* were assessed in five human cancer cell lines.

## Materials and methods

### Reagents

Dihydrotanshinone I, cryptotanshinone and tanshinone I were purchased from Sigma-Aldrich (St. Louis, MO, USA). These reference tanshinones were dissolved in dimethylsulfoxide (DMSO) at a 100 μM concentration and stored at −20°C prior to the experiments, and further dilutions were performed in the culture medium. MTT was obtained from Amresco LLC (Solon, OH, USA) and RPMI-1640 medium, Dulbecco’s modified Eagle’s medium (DMEM), fetal bovine serum (FBS) and penicillin-streptomycin were purchased from HyClone (GE Healthcare, Logan, UT, USA).

### Preparation of crude S. miltiorrhiza fractions

**Figure 1:**
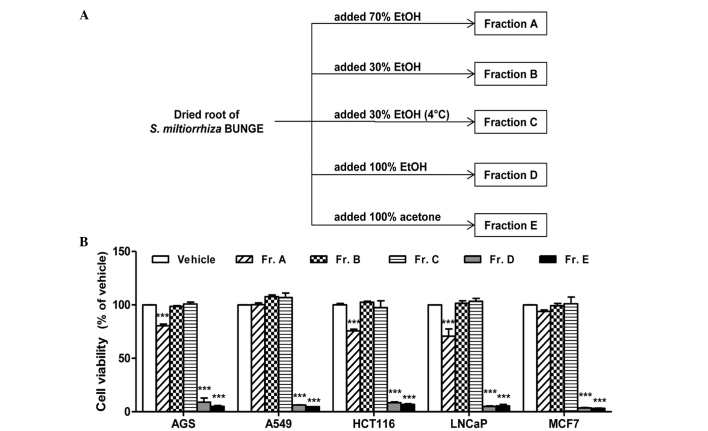
Effect of crude fractions on cell viability. (A) Scheme of crude fraction preparation. (B) Human cancer cells were treated with 250 μg/ml of fractions for 24 h, and then cell viability was determined using the MTT assay. Results are expressed as the mean ± standard error of the mean (n=3). ^***^P<0.001 vs. vehicle-treated control. *S. miltiorrhiza, Salvia miltiorrhiza*; Fr., fraction; EtOH, ethanol.

Extraction was conducted at room temperature by placing 50 g powdered *S. miltiorrhiza* root (Hangzhou Botanical Technology Co., Ltd., Hangzhou, China) in a 500-ml conical flask and adding 250 ml acetone or 100, 70 or 30% aqueous ethanol (v/v). In addition, an extraction using 30% aqueous ethanol at 4°C was conducted. The mouth of the conical flask was covered with aluminum foil and the contents left for 4 h, allowing extraction of the active components into the solvent. Next, the extracts were separated from the residues by filtering the extraction mixture through Whatman No. 1 filter paper (GE Healthcare Life Sciences, Pittsburgh, PA, USA), and the solvent was removed using a rotary vacuum evaporator (Centra Evaporator; Bioneer, Daejon, Korea). Finally, the dried crude extracts were collected and used for further experiments, subsequently labeled fractions A–E (Fig. 1A). The total extract yields were calculated as the percentage weight of extract per 100 g *S. miltiorrhiza* root on a dry basis. These indicated that *S. miltiorrhiza* root extracts obtained using 30% ethanol gave the highest total extract yield (14.5%), followed by 30% ethanol at 4°C (11.7%), 70% ethanol (8.2%), 100% ethanol (0.65%) and acetone (0.58%).

### Cell culture

Human gastric adenocarcinoma (AGS), prostate carcinoma (LNCaP), breast adenocarcinoma (MCF7), colorectal carcinoma (HCT116) and lung adenocarcinoma (A549) cells were purchased from the American Type Culture Collection (Manassas, VA, USA). The AGS, LNCaP and A549 cells were cultured in RPMI-1640 medium with 10% FBS. HCT116 and MCF7 cells were cultured in DMEM with 10% FBS. In addition, the media contained penicillin (100 U/ml) and streptomycin (100 μg/ml). Cells were maintained in a humidified incubator with 5% CO_2_ at 37°C. Cells were subcultured every two days, and cells in the logarithmic growth phase were used for experiments.

### Thin-layer chromatography (TLC) assay

TLC plates (5×10 cm) were prepared by cutting the commercially available sheets (TLC Silica gel 60 F254; Merck, Darmstadt, Germany). The fractions extracted from *S. miltiorrhiza* were transferred and the plates were eluted in a closed chamber with a mobile phase consisting of methylene chloride, methanol and water at a ratio of 25:8:5, respectively. Pure tanshinone reagents, including cryptotanshinone, dihydrotanshinone I and tanshinone I were used as controls.

### Cell cytotoxicity assay

Cells were seeded in 48-well plates at a concentration of 2×10^4^ cells/well and incubated for 24 h at 37°C to allow the cells to adhere to the bottoms of the plates. Cell culture media were removed by aspiration, replaced with fresh media containing an extreme concentration (250 μg/ml) of each fraction, and incubated for 24 h in order to compare the cytotoxic effects of the fractions. Next, the cells were further incubated in the dark with MTT reagent (0.5 mg/ml) for 2 h at 37°C. Subsequently, the MTT reagent-containing culture media was aspirated from each well, and DMSO was added to dissolve the formazan precipitate. The absorbance of each sample was measured using a Multiskan EX microplate reader (Thermo Fisher Scientific, Vantaa, Finland) at a wavelength of 540 nm. Following analysis of the results to determine an appropriate concentration range, the assay was repeated using 0, 5, 10, 20 or 50 μg/ml *S. miltiorrhiza* extract or 0, 1, 5 or 10 μM tanshinone reagent

### Observation of cellular morphology

Following the cell viability assay, cells treated with the three tanshinone reagents were examined for any morphological alterations. Cells were photographed using a Zeiss Axiovert 100 microscope (magnification, ×400; Carl Zeiss Microscopy GmbH, Jena, Germany).

### Statistical analysis

Results are expressed as the mean ± standard error of the mean of three separate experiments. Statistical analyses were performed with GraphPad Prism 5 software for Windows (GraphPad Software, Inc., San Diego, CA, USA). The data were subjected to one-way analysis of variance, followed by Dunnett’s multiple comparison tests. P<0.05 was considered to indicate a statistically significant difference.

## Results

### Influence of solvent and extraction method on the cytotoxic activity of S. miltiorrhiza extracts

The composition and properties of the active constituents in an extract/fraction of plant material vary in response to extraction conditions, including the solvent, extraction time and temperature. Thus, the selection of the extraction method depends on the physical and chemical characteristics of the constituents being investigated. In the present study, cell viability was measured in the presence of an extreme concentration of each fraction (250 μg/ml), in order to compare the cytotoxic effects of the fractions. Fractions D (100% ethanol) and E (100% acetone) exhibited the most notable cytotoxic effect in all five cell lines compared with fractions A–C (Fig. 1B). Among the three fractions extracted with aqueous ethanol solvent (fractions A–C), only fraction A (70% ethanol) produced a moderate reduction in cell viability; however, this effect was limited to the AGS, HCT116 and LNCaP cells. In addition fractions B and C, which were extracted using identical solvents but at different extraction temperatures, exhibited no significant differences in cytotoxicity.

### Effect of ethanolic S. miltiorrhiza extracts on cell viability

**Figure 2:**
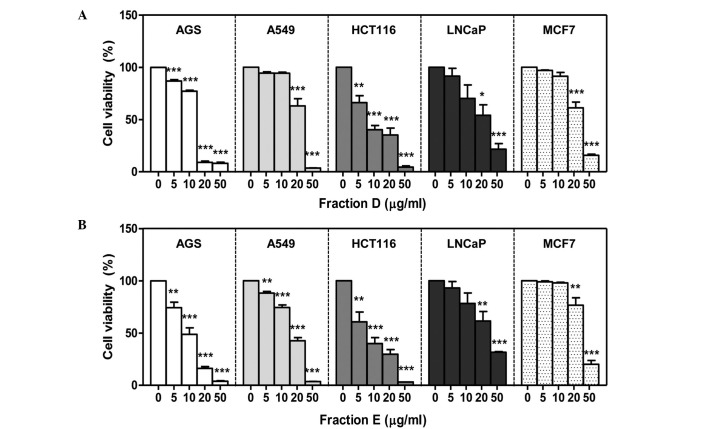
Effect of active fractions on cell viability. Human cancer cells were treated with various concentrations of fractions (A) D and (B) E for 24 h, and cell viability was determined using an MTT assay. ^*^P<0.05, ^**^P<0.01 and ^***^P<0.001 vs. vehicle-treated control.

Next, the concentration-dependent effects of the two most active fractions (D and E) on cancer cell viability were investigated. No differences were observed in the results of the MTT assays conducted using concentrations of fractions D and E ranging from 0 to 250 μg/ml. Thus, 50 μg/ml was the highest concentration investigated for determining the cytotoxic effects of fractions D and E (data not shown). Cells were treated with various concentrations of fractions D or E and reductions of cell viability were identified using an MTT assay. All five tumor cell lines exhibited reductions in cell viability following a 24-h treatment with fractions D or E (Fig. 2). AGS and HCT116 cells were the most affected by the *S. miltiorrhiza* extracts. The IC_50_ values (inhibitory concentration that reduces cell viability by 50%) of fractions D and E in the HCT116 cells were 10.22 and 8.70 μg/ml, respectively (Table I). Compared with the other four cell cancer cell lines, LNCaP cells exhibited lower sensitivity to the cytotoxic effects of fractions D and E. In addition, although the IC_50_ values of fractions D and E in the MCF7 cells were comparable with those in LNCaP cells, a significant cytotoxic effect was only observed in MCF7 cells at concentrations of 20 and 50 μg/ml (Table I; Fig. 2).

### Confirmation of the presence of the reference tanshinones in the active fractions of S. miltiorrhiza

**Figure 3:**
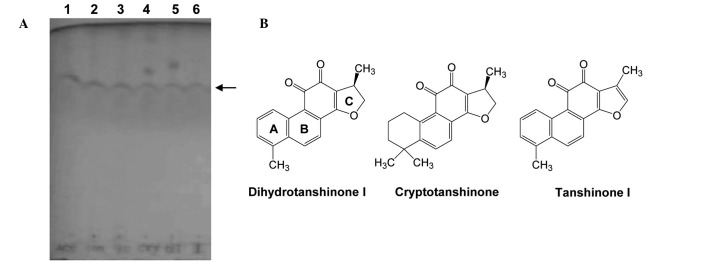
Thin-layer chromatography (TLC) analysis of fractions and tanshinones from *Salvia miltiorrhiza* (Bunge). (A) TLC of fractions D and E of *S. miltiorrhiza*. Lane 1, fraction E; lanes 2 and 3, fraction D; lane 4, cryptotanshinone; lane 5, dihydrotanshinone I; and lane 6, tanshinone I. (B) Chemical structures of reference tanshinones.

Qiu *et al* previously reported that the ethanol extract of *S. miltiorrhiza* is rich in lipophilic constituents, including cryptotanshinone, tanshinone I and IIA, and dihydrotanshinone I (19). The present study investigated whether the ethanol- and acetone-extracted fractions of *S. miltiorrhiza* contained lipophilic tanshinones that were able to produce a reduction in cell viability. The presence of tanshinones in the fractions was initially confirmed by TLC assays, using purified dihydrotanshinone I, cryptotanshinone and tanshinone I as reference materials. Fractions D and E presented bands at similar positions (Fig. 3A). Furthermore, it was observed that the TLC bands of fractions D and E were similar to those of the reference tanshinones (structures presented in Fig. 3B). These results suggest that dihydrotanshinone I, cryptotanshinone and tanshinone I may be the primary constituents of the potent cytotoxic fractions of *S. miltiorrhiza* root.

### Effects of reference tanshinones on the viability of human cancer cells

**Figure 4:**
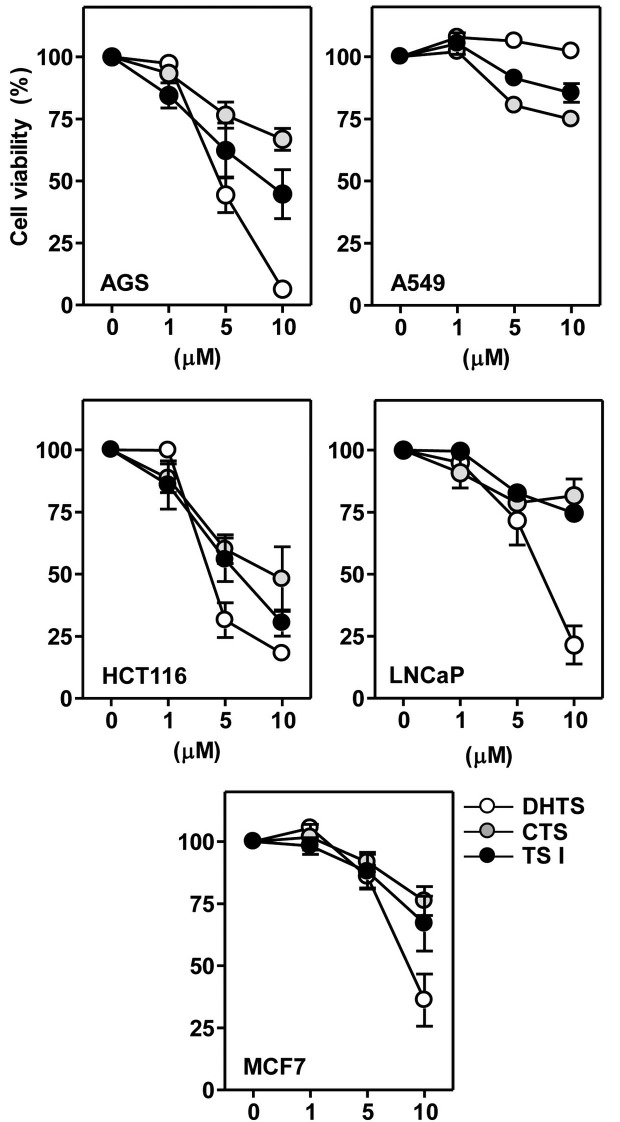
Cytotoxic effect of tanshinones on human cancer cell lines. The cells were treated with different concentrations of DHTS, CTS and TS I for 24 h, and then percentage of cell survival was determined using the MTT assay. Results are expressed as the mean ± standard error of the mean (n=3). DHTS, dihydrotanshinone I; CTS, cryptotanshinone; TS I, tanshinone I.

Next, it was determined whether the reference tanshinones alone were able to affect the viability of cancer cells. Cells were incubated with 1, 5 or 10 μM concentrations of the three tanshinones for 24 h. All three of the examined tanshinones produced reductions in cell viability in a concentration-dependent manner; however, the extent of these effects varied (Fig. 4). Table II presents the IC_50_ values of dihydrotanshinone I, tanshinone I and cryptotanshinone in various cancer cell lines. The IC_50_ values of the tanshinones in A549 cells were not determinable. In all five cancer cell lines, the IC_50_ value and cytotoxic potency of the three tanshinones descended in the following order: Dihydrotanshinone I > tanshinone I > cryptotanshinone (Table II). Among the three tanshinones investigated, dihydrotanshinone I reduced the cell viability with similar potency in the majority of cancer cell lines, with the exception of A549.

### Tanshinone-induced alteration of cellular morphology

**Figure 5:**
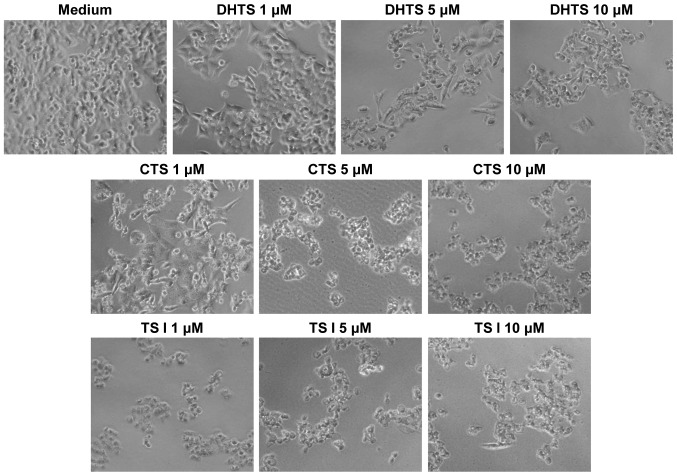
Effect of tanshinones on cell morphology. HCT116 cells were treated with different concentrations of DHTS, CTS and TS I for 24 h, and cell morphology was observed and photographed by phase contrast microscopy (magnification, ×400). CTS, cryptotanshinone; DHTS, dihydrotanshinone I; TS I, tanshinone I.

The effects of the reference tanshinones on cellular morphology were observed directly using an optical microscope. Untreated HCT116 cells were homogeneously distributed on a cultured field, exhibiting a uniform polygonal shape (Fig. 5). Following incubation with the three tanshinones, various morphological changes were observed. Exposure of the cells to tanshinones transformed the shapes of the cells from polygonal to circular, and resulted in cell shrinkage. Furthermore, a reduction in cell number was observed and numerous floating cells were detected. Notably, these morphological alterations were observed at tanshinone concentrations as low as 1 μM. Similar modifications in morphology were observed in the AGS, LNCaP and MCF7 cells following incubation with the tanshinones (data not shown).

## Discussion

The primary objective of the present study was to determine whether extracts of *S. miltiorrhiza* obtained with different solvents differ in their ability to induce cytotoxicity in five human cancer cell lines. Tanshinones, including cryptotanshinone, tanshinone I and IIA, and dihydrotanshinone I, have been reported to be the primary constituents of the ethanol extract of *S. miltiorrhiza,* and to exhibit the most marked cytotoxic effects (19). In addition, the present study investigated the possibility that these tanshinones were responsible for the cytotoxic activity of the ethanol extract of *S. miltiorrhiza*.

The results of the present study indicate that *S. miltiorrhiza* extract, obtained by extraction with 100% ethanol, effectively inhibits cancer cell growth. Solvent-based extraction methods may be advantageous compared with other methods of extracting active components from plant material due to their low cost and simplicity. Traditionally, numerous biologically active compounds have been extracted from plant materials using organic solvents, such as hexane, ether, acetonitrile, benzene and ethanol, at various dilutions (20,21). However, these solvents may exert toxic effects in human patients, limiting their therapeutic potential (22). Thus, the solvent must be separable from the final extract, particularly if the product is intended to be used in food applications (21). Ethanol is a typical solvent used for plant extraction and is safe for human consumption. Ethanol is considered to be ‘generally recognized as safe’ by the US Food and Drug Administration designation for food additives, functional foods and dietary supplements (23). In the Republic of Korea particularly, ethanol is recommended for the preparation of food-grade extracts according to ‘Regulation on approval of functional food ingredients for health functional food’ (24). The results of the present study suggest that the ethanol extract of *S. miltiorrhiza* may be used as a functional food ingredient or as a dietary supplement for the enhancement of health.

In the present study, fraction D (100% ethanol) exhibited the most potent cytotoxic activity, comparable to that of the acetone extract (fraction E; Fig. 1B). It is widely accepted that the specific components responsible for the anti-cancer activity of *S. miltiorrhiza* are diterpenoids with a furano-1,2- or a furano-1,4-naphthoquinone skeleton, otherwise known as tanshinones. A previous study demonstrated that the ethanol extract of *S. miltiorrhiza* root contained 729.6 μg dihydrotanshinone I, 352.4 μg cryptotanshinone, 88.95 μg tanshinone I and 649.9 μg tanshinone IIA per gram of dried root (14). This may explain why fraction D produced the most notable reduction in the growth of cancer cells. However, further research is required in order to identify the chemical compositions of the individual *S. miltiorrhiza* extracts.

In the present study, differences in the efficacy of the extracts obtained using ethanol as a solvent at 30, 70 and 100%, suggested that the concentration of ethanol used affected the cytotoxic activity of the resulting extract. The 100% ethanol extract of *S. miltiorrhiza* exhibited the most marked cytotoxic effect, followed by 70 and 30%. Previous studies have reported that the hydrophilic compounds present in *S. miltiorrhiza* exhibit anti-cancer activity in a number of tumor cell types (25–27). However, in the present study, the 30% ethanol extract (fraction B) was unable to induce toxic effects in any of the five cancer cell lines. It remains unclear why the 30% ethanol extract of *S. miltiorrhiza* was only minimally toxic to the cancer cells. The possibilities include the potency or selectivity of hydrophilic compounds in the extract. For example, protocatechualdehyde in the aqueous fraction of *S. miltiorrhiza* reduced cell growth of HCT116 cells by 21% at 50 μM (28), while the IC_50_ values of the tanshinones in the present study were <10 μM in the same cell lines. The concentration of biologically active hydrophilic compounds in the 30% ethanol extract is another possible reason for the lack of observed cytotoxicity. Thus, there is a requirement for further studies to elucidate the chemical composition and concentrations of the active constituents of the ethanol extracts of *S. miltiorrhiza*.

To date, ~1,700 studies have been published on *S. miltiorrhiza*, <200 in association with cancer and more than half concerning tanshinones. Among the numerous compounds previously identified in *S. miltiorrhiza*, tanshinones have become increasingly studied due to their relatively high abundance and their ability to exert a cytotoxic effect by inhibiting growth and inducing apoptosis. In the present study, dihydrotanshinone I exhibited the most notable cytotoxicity against the human cancer cell lines, followed by tanshinone I and cryptotanshinone. These results are consistent with previous reports, which indicated that dihydrotanshinone I possesses the most marked cytotoxic effect among the three tanshinones examined in numerous human cancer cell lines (14,29,30). As these three tanshinones possess similar chemical structures, the limited variations in these structures may be responsible for the differences in their cytotoxic effects. As presented in Fig. 3B, the only structural differences among the three reference tanshinones are in aromatic rings A and C. A previous report indicated that the structure of ring A may contribute to the cytotoxic effect, resulting in the increased cytotoxic potential of dihydrotanshinone I and tanshinone I compared with cryptotanshinone, in terms of growth inhibition (29).

There are a limited number of studies regarding the effects of the active components of *S. miltiorrhiza* in humans. In China, *S. miltiorrhiza* is used extensively in various TCM preparations, and ~80,000,000 kg of crude *S. miltiorrhiza* extract is consumed as a drug every year (31). The long historical use of this plant as a TCM indicates that *S. miltiorrhiza* produces limited side-effects and may be safe for human consumption. Studies of the anti-cancer potential of *S. miltiorrhiza* began in the early 1990s, although it has been used for >2,000 years for medicinal purposes (32,33). Studies published in the two subsequent decades have reported that *S. miltiorrhiza* extracts and tanshinones possess broad-range growth inhibitory activity against various cancer cell lines (34,35). Furthermore, two reports have indicated that combinations of *S. miltiorrhiza* extracts with other medicinal plants modulate immunological functions in patients (36,37). Capsules containing *S. miltiorrhiza* combined with the mushroom *Trametes versicolor* (known as yunzhi in China) appeared to alleviate lymphopenia to patients with nasopharyngeal carcinoma, when administered orally for 16 weeks during the course of radiotherapy (36). In patients with breast cancer, regular oral consumption of these capsules was observed to promote cell-mediated and humoral immunological functions, and thus exhibited an anti-cancer effect (37). No serious adverse side-effects were observed in these studies. Based on these previous studies, we hypothesize that *S. miltiorrhiza* may be used safely as an adjunct treatment. Thus, previous studies suggest that the consumption of the ethanol extract of *S. miltiorrhiza* as an ingredient in functional foods need not be limited to healthy individuals but may aid cancer patients and enhance the efficacy of standard cancer therapy.

In conclusion, the present study indicates that the ethanol extract of *S. miltiorrhiza* inhibits the growth of cancer cells. Furthermore, the tanshinone components in this extract appear to be capable of effectively reducing cancer cell viability. These results support the use of the ethanol extract of *S. miltiorrhiza* as a novel, efficacious and safe candidate for dietary supplements or as an ingredient in functional foods. However, future studies, involving clinically relevant animal models, are required to further elucidate the potential of *S. miltiorrhiza* extract as a therapeutic agent.

## Figures and Tables

**Table I:** Cytotoxic effect of extract fractions D and E on five human cancer cell lines (IC_50_, *μ*g/ml).

Fraction	AGS	A549	HCT116	LNCaP	MCF7
D	10.44	15.72	10.22	22.65	22.15
E	9.22	13.64	8.70	29.86	25.50

**Table II:** Cytotoxic effect of tanshinones from *Salvia miltiorrhiza* (Bunge) on five human cancer cell lines (IC_50_, μM).

Reagent	AGS	A549	HCT116	LNCaP	MCF7
DHTS	3.35	>10	3.87	5.29	8.02
CTS	>10	>10	8.84	>10	>10
TS I	8.21	>10	5.82	>10	>10

DHTS, dihydrotanshinone I; CTS, cryptotanshinone; TS I, tanshinone I.
